# Relationship between psychological contract fulfillment, job burnout, and job satisfaction among pharmacists in private medical institutions in Guiyang, China

**DOI:** 10.3389/fpubh.2025.1641543

**Published:** 2025-08-04

**Authors:** Ting Zhang, Man Ao, Lei Lu, Shuya Chen, Yongyong Luo, Fushan Tang

**Affiliations:** ^1^Department of Pharmacy, Guiyang Hospital of Stomatology, Guiyang, China; ^2^Department of Pharmacy, The First People’s Hospital of Guiyang, Guiyang, China; ^3^Department of Clinical Pharmacy, Key Laboratory of Basic Pharmacology of Guizhou Province and School of Pharmacy, Zunyi Medical University, Zunyi, China; ^4^Key Laboratory of Basic Pharmacology of Ministry of Education and Joint International Research Laboratory of Ethnomedicine of Ministry of Education, Zunyi Medical University, Zunyi, China; ^5^Key Laboratory of Clinical Pharmacy in Zunyi City, Zunyi Medical University, Zunyi, China

**Keywords:** psychological contract, job burnout, job satisfaction, private medical institutions, pharmacist

## Abstract

**Objective:**

This research aims to systematically investigate the mechanisms between psychological contract fulfillment and job burnout/job satisfaction among pharmacists, addressing the research gap in this population.

**Methods:**

This is a cross-sectional research that employed convenience sampling to recruit 384 pharmacists from private medical institutions in Guiyang between May and August 2024. The research utilized scales for psychological contract, job burnout, and job satisfaction for analysis. After conducting reliability and validity tests on the questionnaires, correlation analysis, mediation analysis, and multiple linear regression analysis were employed to explore the relationships among psychological contract fulfillment, job burnout, and job satisfaction among pharmacists in private medical institutions.

**Results:**

The findings indicate that pharmacists’ psychological contract fulfillment is significantly weakly negatively correlated with job burnout (*r* = −0.187, *p* < 0.01) and significantly strongly positively correlated with job satisfaction (*r* = 0.528, *p* < 0.01), and a significant strongly negatively correlation between the job burnout and the job satisfaction (*r* = −0.436, *p* < 0.01). Mediation analysis shows that job burnout plays an weakly mediating role in the relationship between psychological contract fulfillment and job satisfaction (12.16%, *p* < 0.01). This suggests that burnout is merely a minor component within a much broader context. In addition, a considerable proportion of pharmacists 43.23% indicated that their level of psychological contract fulfillment did not meet the benchmark value (most of the responsibility has been fulfilled). indicating significant psychological contract breaches within this group. Multiple linear regression analysis further identifies that demographic variables (age, employment length), psychological contract fulfillment, and burnout levels collectively serve as core predictors of job satisfaction.

**Conclusion:**

The research suggests that private medical institutions should take proactive measures to ensure the stability of pharmacists’ psychological contracts. For example, during the intervention process, preventive guidance should be provided to senior pharmacists to help them avoid breaches of the psychological contract and the further development of job burnout. For newly recruited pharmacists, selective preventive interventions should be implemented to achieve personalized psychological contract interventions. Finally, this research helps fill the research gap regarding pharmacists’ psychological contracts in private medical institutions.

## 1 Introduction

Private medical institutions play a crucial role in China’s medical system, forming a comprehensive service network alongside public institutions (1, 2). According to the China Health Statistics Yearbook (2012–2021), the per capita density of private medical institutions in Guizhou Province is higher than in other regions, highlighting the importance of prioritizing the development of these institutions (3). Additionally, in 2024, the number of private medical institutions in Guizhou reached 1,261, accounting for 80.7% of the total number of medical institutions (with only 302 Public medical institutions) (4). This is due to Guizhou’s unique position in the medical supply system and the special challenges it faces. As an important province in southwestern China, Guizhou’s medical resources are unevenly distributed, and grassroots medical conditions are relatively underdeveloped. Private medical institutions play a key role in compensating for the shortage of resources in medical institutions and meeting diverse medical needs. Pharmacists, as guardians of rational drug use in medical institutions, are an indispensable part of medical institutions development, but the value of pharmacists in private medical institutions is often underestimated (5). There is an unreasonable discrepancy between their salary structure and clinical responsibilities. Compared to the workload, the average salary is still too low, and there is a lack of performance-based incentives (6). This gap not only reduces job satisfaction but also weakens staff retention rates. Moreover, the shortage of pharmacists in private medical institutions forces them to take on extensive responsibilities. This work overload leads to emotional stress and job burnout (7, 8), and the lack of standardized career development paths severely hinders the career planning and promotion of pharmacists (9, 10). These issues stem from significant differences between private institutions and public medical institutions in terms of management mechanisms, salary systems, and career development paths. Research have shown that employees in private institutions generally earn lower salaries than those in public medical institutions and lack clear career advancement channels, which directly lead to difficulties in fulfilling psychological contracts, resulting in high incidences of job burnout. A psychological contract refers to the implicit, informal expectations and responsibilities between individuals and organizations. It reflects the expectations that employees have of the organization and the commitments that the organization makes to employees (11). Content analysis of the literature shows that psychological contracts are multidimensional, including transactional elements (such as salary and job security) and relational elements (such as career development opportunities and work-life balance regulations) (12). Importantly, these contract dimensions operate within the boundaries of time and context and influence organizational behavior through perceived reciprocity mechanisms (13). Over the past three decades, psychological contract theory has undergone significant evolution (14, 15). This evolution is reflected in the development and refinement of its theoretical framework, clearer content and organizational principles, and extensive research on the behavior following psychological contract violations. Furthermore, the establishment and repair of psychological contracts have also been thoroughly discussed (16). As a management theory and methodology, its application has become more specific and widespread, significantly enhancing organizational management processes. In recent years, the application of psychological contract theory in the medical sector has gradually gained attention. Research have indicated that the degree of fulfillment of psychological contracts is closely related to the job satisfaction and job burnout levels of medical workers. When psychological contracts are well-fulfilled, employees tend to have higher job motivation and satisfaction, while a breach or failure to fulfill the psychological contract leads to dissatisfaction and burnout (17), which in turn leads to decreased job satisfaction, increased turnover rates, and other issues. Symptoms of burnout, such as emotional exhaustion, depersonalization, and reduced personal accomplishment, may occur. Additionally, the fulfillment of psychological contracts not only affects employee job satisfaction but also indirectly influences job burnout through its impact on perceived organizational support and work engagement. Therefore, maintaining and fulfilling psychological contracts is crucial for reducing job burnout, enhancing employee mental health, and improving organizational performance. In private institutions in Guizhou, due to the lack of institutionalized management mechanisms and career development paths, employees’ psychological contracts are often in an unstable state, leading to significantly higher incidences of job burnout and low job satisfaction compared to public medical institutions. In private institutions in Guizhou Province, due to the lack of institutionalized management mechanisms and career development paths, pharmacists’ psychological contracts are often in an unstable state. This leads to a significantly higher incidence of job burnout and lower job satisfaction compared to Public medical institutions. Pharmacists in private medical institutions face prominent issues such as job burnout, unfulfilled psychological contracts, and declining job satisfaction, making them an important sample for studying the psychological state of medical workers. Therefore, this research will focus on exploring the role of psychological contracts in job burnout and job satisfaction among pharmacists in private medical institutions in Guizhou Province, providing theoretical support for relevant policy formulation and human resource management (18).

## 2 Materials and methods

### 2.1 Procedure and data collection

This is a cross-sectional research that employed convenience sampling to private medical institutions pharmacist (19). All participants were fully informed about the nature of the research and their voluntary participation. The participants were primarily pharmacists from private medical institutions. Pharmacists were selected from private medical institutions. Across six districts and counties in Guiyang, ensuring broad coverage of the target population. Data collection took place between May and August 2024. A total of 400 questionnaires were distributed, with 384 valid responses collected, resulting in a response rate of 96%. All responses were anonymized, with personal identifiers removed to ensure privacy.

### 2.2 Psychological contract scale

The psychological contract scale was derived from previous research (20) and consists of three main components, totaling 40 items: 17 items focusing on hospital responsibilities, 14 items on pharmacist responsibilities, and 9 items on social and governmental responsibilities. Each item was rated on a 7-point scale ranging from 0 to 6. “Not the responsibility of the hospital or pharmacist” [0], “the responsibility has not totally been fulfilled” [1], “the responsibility has basically not been fulfilled” [2], “a small part of the responsibility has been fulfilled” [3], “half of he responsibility has been fulfilled” [4], “most of the responsibility has been fulfilled” [5], and “all the responsibility has been fulfilled” [6].

### 2.3 Job burnout scale

**Table:** 

Dimension	Number	Scoring range	Scoring meaning
Emotional exhaustion	9	0 (never) – 6 (every day)	Scores above 27 indicate high emotional exhaustion, while scores below 16 indicate low emotional exhaustion.
Depersonalization	5	0 (never) – 6 (every day)	Scores above 9 signify high depersonalization, and scores below 6 indicate low depersonalization.
Personal accomplishment	8 (21)	0 (never) – 6 (every day) (22)	Scores above 39 suggest high personal accomplishment, whereas scores below 31 indicate low personal accomplishment.

The Chinese version of the Maslach Burnout Inventory-Human Services Survey (MBI-HSS), originally developed by Maslach et al. (23) and revised by Li et al. (24).

### 2.4 Job satisfaction scale

The job satisfaction scale was developed through a review of relevant literature and interviews with pharmacists from private medical institutions (25). It was used after conducting preliminary research. The scale evaluates job satisfaction across seven dimensions: job autonomy, work environment, scheduling, communication opportunities, recognition, growth and development, and compensation and benefits (26). Each item is rated on a 5-point scale ranging from 1 (strongly dissatisfied) to 5 (strongly satisfied).

### 2.5 Reliability and validity analysis

Reliability and validity of the scales used in this research were assessed through exploratory factor analysis and reliability testing. A Kaiser-Meyer-Olkin (KMO) value greater than 0.5 and a Cronbach’s alpha greater than 0.8 were considered indicative of good reliability and validity (27, 28).

### 2.6 Statistical analysis

Data entry and statistical analysis were conducted using IBM Statistical Package for the Social Sciences (SPSS) version 26.0. The research employed descriptive analysis, correlation analysis, mediation effect analysis, and linear regression analysis to examine the relationships between the variables under investigation, Mediation analysis was conducted using multiple regression equations to empirically test the mediating role of the mediator variable in the pathway between the independent and dependent variables.

## 3 Results

### 3.1 Demographic data

In the valid questionnaires, there were 93 males and 291 females. Age groups: 21–30 years old:144, 31–40 years old:158, 41–50 years old:47, 51–60 years old:32, and over 60 years old:3. Education: Technical secondary school:3, Two (or three) year college:101, Undergraduate:271, Graduate or above:9. Pharmacy background: 347 and non-pharmacy background:37. Hospital grade: Level 3:109, Level 2:150, Level 1:97, Other:20. Professional titles: None:22, Pharmacist:223, Pharmacist in charge:122, Associate chief pharmacist:17. Monthly salary: 1000–1999 CNY:5, 2000–2,999 CNY:34, 3,000–3,999 CNY: 105, 4,000–4,999 CNY:97, Above 5,000 CNY:143. Hospital category: General hospital: 244, Specialized hospital:140. Employment length (years): Less than 1 year:51, 1–2 years:65, 3–5 years:99, Over 5 years:169. The demographic characteristics of the participants are shown in Table 1. The impact of various demographic factors on psychological contract, job burnout, and job satisfaction is shown in Table 2.

**Table 1:** Demographic statistics for the sample.

Characteristic		Frequency	Percentage (%)	Characteristic		Frequency	Percentage (%)
Sex	Male	93	24.22	Monthly salary (CNY)	1,000–1999	5	1.31
	Female	291	75.78		2000–2,999	34	8.85
Age	21–30	144	37.50		3,000–3,999	105	27.34
	31–40	158	41.15		4,000–4,999	97	25.26
	41–50	47	12.24		≥5,000	143	37.24
	51–60	32	8.33	Position	Associate director and above	35	9.11
	60-	3	0.78		Department head	87	22.66
Education	Technical secondary school	3	0.78		None	262	68.23
	Two (or three)year college	101	26.31	Marital status	Married	269	70.05
	Undergraduate	271	70.57		Unmarried	99	25.78
	Graduate or above	9	2.34		Divorce or widowhood	16	4.17
Professional title	None	22	5.73	Employment length (years)	Less than 1 year	51	13.28
	Pharmacist	223	58.07		1–2 years	65	16.93
	Pharmacist in charge	122	31.77		3–5 years	99	25.78
	Associate chief pharmacist	17	4.43		Over 5 years	169	44.01
Academic background	Pharmacy	347	90.36	Hospital category	General hospital	244	63.54
	Non-pharmacy	37	9.64		Specialized hospital	140	36.46
Hospital grade	Level 1	97	25.26				
	Level 2	150	39.06				
	Level 3	109	28.39				
	Other	28	7.29				

**Table 2:** Demographic factors on psychological contract, burnout, and satisfaction.

Characteristic		Psychological contract	*p*	Burnout	*p*	Satisfaction	*p*
Sex	1	197.10 (34.31)	0.509	45.81 (29.62)	0.327	25.85 (5.36)	0.252
	2	194.41 (34.10)		42.53 (21.83)		25.15 (4.17)	
Age	1	199.33 (34.02)	0.241	40.03 (23.17)	0.215	26.26 (4.81)	0.019*
	2	193.68 (33.17)		46.06 (25.21)		25.00 (4.32)	
	3	186.85 (40.26)		45.91 (20.76)		24.49 (3.63)	
	4	193.84 (28.07)		41.47 (25.14)		23.97 (4.46)	
	5	204.33 (34.96)		36.33 (14.74)		24.67 (3.51)	
Education	1	200.33 (35.73)	0.774	31.00 (7.00)	0.065	25.00 (5.20)	0.654
	2	197.90 (33.50)		38.83 (20.65)		25.35 (4.42)	
	3	193.88 (34.77)		44.77 (24.81)		25.38 (4.56)	
	4	196.89 (21.44)		54.22 (28.90)		23.44 (3.05)	
Professional title	1	203.45 (34.48)	0.153	46.95 (23.09)	0.042*	25.91 (5.21)	0.522
	2	197.33 (33.77)		40.71 (24.11)		25.52 (4.57)	
	3	189.97 (34.48)		47.97 (23.49)		24.84 (4.36)	
	4	191.00 (33.52)		39.53 (22.42)		25.35 (3.33)	
Academic background	1	194.54 (34.50)	0.360	43.50 (23.94)	0.666	25.30 (4.60)	0.754
	2	199.95 (30.45)		41.70 (24.29)		25.54 (3.36)	
Hospital grade	1	194.67 (33.21)	0.682	50.19 (28.60)	0.003*	25.04 (4.42)	0.838
	2	192.96 (35.47)		43.11 (21.43)		25.37 (4.24)	
	3	198.20 (31.84)		39.73 (23.88)		25.36 (5.10)	
	4	195.43 (39.23)		34.64 (10.58)		25.89 (3.60)	
Monthly salary (CNY)	1	162.40 (77.39)	0.036*	52.00 (20.46)	0.729	25.00 (7.91)	0.001*
	2	193.03 (37.06)		45.62 (20.77)		24.71 (4.58)	
	3	194.26 (39.99)		43.52 (24.60)		25.30 (4.30)	
	4	190.32 (33.89)		44.54 (19.95)		23.98 (4.34)	
	5	200.49 (24.94)		41.50 (26.73)		26.41 (4.36)	
Position	1	199.06 (23.05)	0.108	47.89 (28.76)	0.390	24.66 (3.45)	0.523
	2	188.38 (36.61)		44.43 (21.68)		25.10 (4.29)	
	3	196.74 (34.33)		42.35 (23.98)		25.48 (4.68)	
Marital status	1	193.54 (35.07)	0.096	42.93 (22.55)	0.794	25.12 (4.25)	0.097
	2	200.80 (30.87)		43.81 (27.69)		26.07 (4.93)	
	3	185.19 (34.29)		46.88 (23.12)		24.00 (5.18)	
Employment length (years)	1	202.98 (31.91)	0.132	41.53 (23.39)	0.617	26.02 (4.25)	0.000*
	2	198.91 (28.80)		43.17 (27.49)		25.97 (4.24)	
	3	194.83 (34.66)		41.40 (24.84)		26.57 (5.68)	
	4	191.33 (36.01)		45.05 (22.16)		24.13 (3.50)	
Hospital category	1	194.71 (32.96)	0.791	42.87 (25.21)	0.624	25.44 (4.75)	0.482
	2	195.67 (36.18)		44.11 (21.65)		25.11 (4.00)	

### 3.2 Testing for reliability and validity

The Cronbach’s alpha coefficients for the psychological contract scale, job burnout scale, and job satisfaction scale were 0.973, 0.931 and 0.922 respectively, demonstrating excellent internal consistency across all measurement tools. To assess the factor structure validity, exploratory factor analysis (EFA) was conducted with the Kaiser-Meyer-Olkin (KMO) measure and Bartlett’s test of sphericity. KMO: >0.50 indicates suitability for factor analysis. Bartlett’s test of sphericity: *p* < 0.05, indicates suitability for factor analysis. The research results show KMO values reached 0.954 for psychological contract, 0.936 for job burnout, and 0.914 for job satisfaction, all exceeding the recommended threshold of 0.5, while Bartlett’s tests of sphericity were statistically significant (*p* < 0.001), indicating adequate sampling adequacy and factor ability of the correlation matrices (29). All three scales demonstrate good reliability and validity.

### 3.3 Psychological contract

In comparing the three dimensions of psychological contracts, statistically significant differences were observed among these dimensions. Using “most of the responsibility has been fulfilled” as the threshold criterion (5 score*40 items = 200), 43.23% of pharmacists perceived an overall inadequate fulfillment level of psychological contracts. Specifically, hospital responsibilities received relatively lower ratings compared to pharmacists’ responsibilities and government/social responsibilities. This indicates that most pharmacists have a more urgent demand for improvement in the fulfillment of hospital responsibilities, shown in Table 3.

**Table 3:** Analysis of the dimensions of psychological contract.

Items	Hospital	Pharmacist	Government/Society
	73.89 (16.42)	75.86 (12.30)	45.30 (10.14)
*F*		553.09	
*p*		0.000**	
Psychological contract score	<200		≥200
*N* (%)	166 (43.23)		218 (56.77)

**p < 0.01.

### 3.4 Job burnout

Pharmacists in private medical institutions exhibit moderate emotional exhaustion ( $\bar{x}$ ) 16.40 >16 score, low personal accomplishment ( $\bar{x}$ ) 19.71 <31 score, and moderate depersonalization ( $\bar{x}$ ) 7.21 >6 score, collectively contributing to an intermediate level of overall burnout, shown in Table 4.

**Table 4:** Analysis of the dimensions of job burnout.

Items	Category/Option	*N*	%	$\bar{x}$ (SD)
Emotional exhaustion	Minimum–Maximum		0–54	16.40 (11.98)
	Low	213	55.47	
	Moderate	141	36.72	
	High	30	7.81	
Personal accomplishment	Minimum–Maximum		0–42	19.71 (9.23)
	Low	361	60.68	
	Moderate	7	33.33	
	High	16	5.99	
Depersonalization	Minimum–Maximum		0–30	7.21 (7.19)
	Low	97	25.26	
	Moderate	61	15.89	
	High	226	58.85	

### 3.5 Pharmacists’ job satisfaction

Regarding the job satisfaction of pharmacists in private medical institutions, using “relatively satisfied” as the baseline 28 (4 score*7 items = 28), over 59.11% of pharmacists reported poor job satisfaction. The lowest-scoring item was compensation and benefits (3.35), with average scores generally falling between 3 and 4. This suggests that the overall satisfaction of pharmacists in private medical institutions ranges from neutral to relatively satisfied, indicating there is still substantial room for improvement, shown in Table 5.

**Table 5:** Analysis of pharmacists’ job satisfaction.

Items	$\bar{x}$ (SD)	
Work autonomy/work responsibility balance (basic workload, colleagues competence, clear task allocation, work pressure)	3.69 (0.738)	
Work environment (employee working conditions, safety, facilities and staffing)	3.71 (0.735)	
Scheduling (number of working days, daily working hours)	3.64 (0.787)	
Opportunities for communication (interaction with colleagues within and outside the department, communication with leadership)	3.65 (0.764)	
Opportunities for praise/recognition (recognition from patients and colleagues, acknowledgment from leadership, sense of accomplishment)	3.70 (0.703)	
Growth and development (participation in research, promotion opportunities, skill training)	3.59 (0.835)	
Compensation and benefits (social insurance, housing fund, holidays, salary)	3.35 (0.870)	
Job satisfaction total score	<28	≥28
*N* (%)	227 (59.11)	157 (40.89)

### 3.6 Correlation analysis

The correlation analysis showed a significant weakly negatively correlation between the total psychological contract score and the total job burnout score (*r* = −0.187, *p* < 0.01), a significant strongly positively correlation between the total psychological contract score and the total job satisfaction score (*r* = 0.528, *p* < 0.01), and a significant strongly negatively correlation between the total job burnout score and the total job satisfaction score (*r* = −0.436, *p* < 0.01). These results indicate that positive feedback from the psychological contract can directly enhance pharmacists’ job satisfaction, whereas negative feedback from job burnout can directly reduce their job satisfaction, as shown in Table 6.

**Table 6:** Correlation analysis of the variables.

Variables	$\bar{x}$ (SD)	1	2	3	4	5	6	7	8	9
Hospital	73.89 (16.42)	1								
Pharmacist	75.86 (12.30)	0.595**	1							
Government/society	45.30 (10.14)	0.674**	0.707**	1						
Psychological contract	195.06 (34.13)	0.896**	0.857**	0.876**	1					
Emotional exhaustion	16.40 (11.98)	−0.165**	−0.139**	−0.147**	−0.173**	1				
Personal accomplishment	19.71 (9.23)	−0.121**	−0.172**	−0.208**	−0.182**	0.481**	1			
Depersonalization	7.21 (7.19)	−0.129**	−0.069	−0.043	−0.100	0.796**	0.374**	1		
Job burnout	43.32 (23.95)	−0.168**	−0.157**	−0.167**	−0.187**	0.925**	0.739**	0.843**	1	
Job satisfaction	25.32 (4.49)	0.506**	0.388**	0.489**	0.528**	−0.393**	−0.435**	−0.238**	−0.436**	1

*p < 0.05, **p < 0.01.

### 3.7 Mediation effect analysis

**Figure 1:**
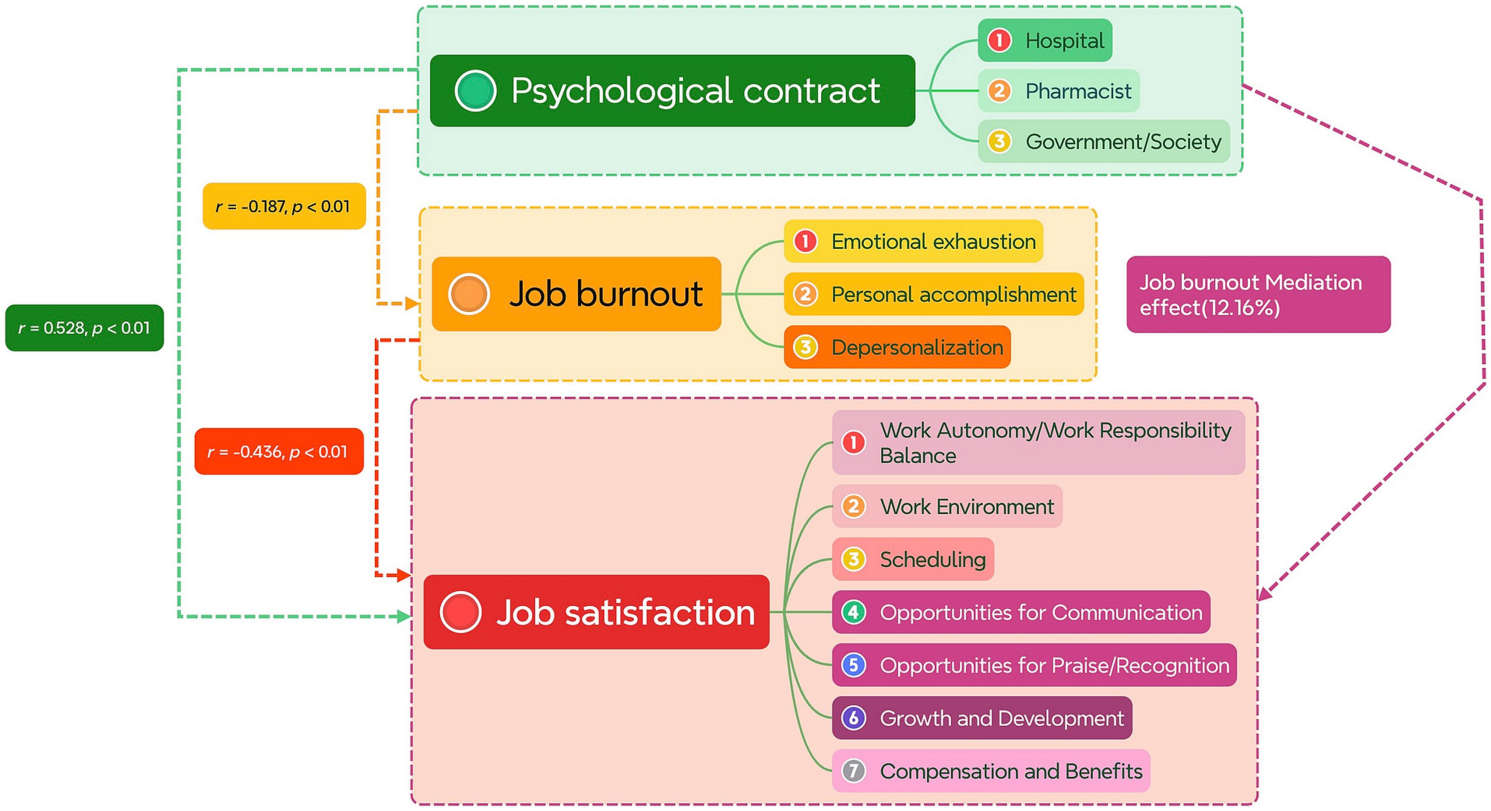
Relationship model diagram.

Mediation analysis was conducted using the mediation test procedure in sequence. First, regression analysis was performed with the total score of the psychological contract as the independent variable and the total score of job satisfaction as the dependent variable, to test whether the regression coefficient between the two reached significance. Then, regression analysis was performed again with the total score of the psychological contract as the independent variable and the total score of job burnout as the dependent variable to test for significance. Finally, regression analysis was conducted with the total score of job satisfaction as the dependent variable, and both the total scores of job burnout and the psychological contract as independent variables, resulting in a mediation effect of (0.131*0.065/0.070*100% = 12.16%). The results indicate that job burnout plays a weak partial mediating role in the relationship between psychological contract and job satisfaction among pharmacists in private medical institutions. Therefore, improving the level of job burnout may enhance the job satisfaction of pharmacists in private medical institutions, as shown in Table 7. The correlation and mediating effect of the three variables can be seen in Figure 1.

**Table 7:** Analysis of the mediating role of job burnout.

Step	Standardized regression equation	Regression coefficient testing
1	Y = 11.763 + 0.070X	t1 = 10.392**, t2 = 12.158**
2	M = 68.913 − 0.131X	t1 = 9.866**, t2 = −3.719**
3	Y = 16.276 + 0.061X − 0.065 M	t1 = 14.014**, t2 = 11.428**, t3 = −8.622**

**p < 0.01, Y: job satisfaction, X: psychological contract, M: job burnout.

### 3.8 Multiple linear regression analysis

After checking for multicollinearity using VIF and ensuring that the residuals meet the normality assumptions, the results of the multiple linear regression analysis, with demographic factors, total psychological contract score, and total job burnout score as independent variables and job satisfaction as the dependent variable, are shown in Table 8.

**Table 8:** Analysis of influencing factors on job satisfaction.

Variables	*B*	beta	*t*	*p*	VIF
Sex	−0.761	−0.073	−1.757	0.080	1.113
Age	−0.695	−0.147	−2.820	0.005**	1.760
Education	−0.153	−0.017	−0.383	0.702	1.306
Professional title	0.696	0.102	1.946	0.052	1.779
Academic background	0.190	0.013	0.311	0.756	1.054
Employment length (years)	−0.414	−0.099	−2.192	0.029**	1.314
Hospital category	−0.377	−0.010	−1.000	0.318	1.067
Hospital grade	−0.185	−0.037	−0.866	0.387	1.179
Monthly salary(CNY)	0.141	0.033	0.733	0.464	1.330
Position	0.054	0.008	0.172	0.863	1.321
Marital status	−0.218	−0.027	−0.634	0.527	1.183
Psychological contract	0.059	0.445	10.892	0.000**	1.087
Job burnout	−0.066	−0.351	−8.421	0.000**	1.130

**p < 0.01.

## 4 Discussion

### 4.1 Demographic data analysis

This research aims to explore the intrinsic connections between psychological contract, job burnout, and job satisfaction. The analysis reveals that monthly salary is the primary factor influencing the psychological contract among pharmacists, which is consistent with reality. Compensation is not only a crucial indicator of labor value but also an intrinsic motivator for pharmacists’ work engagement (30).

Secondly, professional title and medical institutions accreditation level are the main factors contributing to job burnout among pharmacists. Within the medical system, pharmacists with higher professional titles often assume more critical work roles. The prolonged stress associated with these roles poses a significant risk of job burnout. In higher-accredited medical institutions, pharmacists are not only responsible for dispensing medications but also for tasks such as patient education and pharmaceutical care monitoring. These responsibilities require intense mental effort, long-term accumulation of experience, and continuous learning, all of which are highly likely to lead to job burnout.

Lastly, age, monthly salary, and employment length (31) are key factors affecting job satisfaction. Young pharmacists who have recently joined the workforce typically hold positive expectations for their jobs. Their initial psychological contracts are relatively simple, focusing primarily on basic working conditions and compensation benefits. These needs are often met during the early stages of employment, and they usually experience strong organizational support (such as on boarding training). This support enhances their trust in and sense of belonging to the organization, thereby increasing job satisfaction. They are also optimistic about their career development, believing that they have significant room for growth and promotion opportunities within the organization. This positive outlook significantly boosts job satisfaction.

### 4.2 Psychological contract

Taking the fulfillment of most responsibilities as a benchmark, it can be seen that 43.23% of pharmacists in private medical institutions perceive their psychological contract fulfillment as only partially met or halfway fulfilled. Most pharmacists believe that hospital responsibilities are not adequately fulfilled, which is mainly reflected in the following:

Lack of training and promotion mechanisms: Pharmacists’ relational contract expectations regarding career development (such as professional title promotion and skill training) have not been met. Private institutions, driven by economic benefits, have reduced investment in employee development, leading to limited promotion channels (such as insufficient senior title positions and policy discrimination) and formalized continuing education. This violates the “development responsibility” of the organization as stated in the psychological contract (providing long-term growth opportunities).

Dilution of professional value: Pharmacists are forced to take on non-professional roles such as billing and administrative tasks, preventing them from focusing on core pharmaceutical functions (such as medication guidance and clinical collaboration), which leads to a degradation of professional knowledge. This not only weakens their ability to transform technical value into economic value but also undermines the “organizational recognition” element of the psychological contract. In conclusion, the results confirm the core idea of psychological contract theory: the fulfillment of organizational responsibilities is a prerequisite for the fulfillment of employee responsibilities (32).

### 4.3 Job burnout

Pharmacists working in private medical institutions in Guiyang city report experiencing moderate levels of job burnout, a finding that aligns with results from other research (33). Notably, depersonalization (34) stands out as a prominent issue, which may significantly increase the risk of pharmacists developing depression (35). This highlights the critical need to address burnout among pharmacists in these settings, as excessive work pressure and overall job dissatisfaction are likely key contributing factors (36, 37). Numerous research emphasize the importance of prioritizing burnout mitigation and prevention strategies in the workplace to reduce its impact on pharmacists’ performance and prevent turnover (38, 39). Establishing an open communication mechanism, encouraging pharmacists to share the challenges and pressures they face at work with colleagues and management, seeking support and solutions. Management should conduct regular anonymous surveys to assess employee stress levels and adjust policies accordingly. Additionally, group psychological counseling has been proven to effectively reduce pharmacists’ job burnout, enhance their sense of self-efficacy, and improve work efficiency (40).

### 4.4 Job satisfaction

In private medical institutions in Guiyang, 59.11% of pharmacists have a job satisfaction score below 28, indicating that most pharmacists are dissatisfied with their current work. The main areas of dissatisfaction are concentrated in compensation and benefits (41) and growth and development. These findings are consistent with a recent systematic review, which mentioned that burnout, stress, etc., account for 24%, being the primary factors influencing pharmacists’ job satisfaction (42), followed by working conditions, professional development, and earnings and benefits. It can be observed that work conditions, professional development, leadership support, and income benefits are similar to the psychological contract content we studied. These issues may, to some extent, stifle pharmacists’ enthusiasm and creativity, hindering their ability to fully realize their potential. Ultimately, this can negatively impact the stability of the pharmacist workforce and their long-term professional growth.

### 4.5 Correlation analysis and mediation analysis

Research have confirmed that the fulfillment of psychological contracts significantly influences job burnout (43) and job satisfaction among pharmacists in private medical institutions. Job burnout has also been demonstrated as a key factor contributing to turnover (44), while persistently low levels of job satisfaction are likewise a significant driver of pharmacist attrition (45). Prolonged job burnout is more likely to lead to decreased professional fulfillment and job satisfaction among pharmacists (46, 47). The direct impact of psychological contracts on job satisfaction is more significant, suggesting that addressing breaches of psychological contracts can significantly improve job satisfaction. Mediation analysis revealed that job burnout acts as a mediating variable (12.16%), mediating the relationship between psychological contracts and job satisfaction (48). By improving pharmacists’ burnout status, job satisfaction can be indirectly enhanced. However, contrary to research on nurses (49, 50), the mediating effect of job burnout is stronger among nursing staff, although it is higher than that of Chinese primary healthcare staff (5.32%) (51). In conclusion, while the mediating effect of job burnout varies across different groups, it should not be overlooked, and attention should be paid to the mediating role of job burnout in the relationship between pharmacists’ psychological contracts and job satisfaction.

### 4.6 Multiple linear regression analysis

The regression analysis results indicate that the primary factors influencing pharmacists’ job satisfaction include age, employment length, the degree of fulfillment of the psychological contract, and the level of job burnout. This is consistent with other research findings (52). Once pharmacists have the opportunity to learn new skills and gain a sense of career achievement, along with a higher degree of psychological contract fulfillment, their job satisfaction can be improved. However, older pharmacists often engage in repetitive tasks and lack many opportunities for training, which may lead to burnout (53, 54), gradually resulting in a decline in job satisfaction (55). Older pharmacists with longer work experience tend to have lower satisfaction compared to younger pharmacists. Despite their extensive experience, salary increases often fail to keep pace with years of service. Even after many years of service, stagnant pay scales can reduce satisfaction with compensation (56). Other research mention that workload, stress, promotion opportunities, job security, autonomy, and work atmosphere are factors influencing pharmacists’ job satisfaction (57). Long-term workplace interactions can also lead to conflicts with colleagues, supervisors, or patients, further negatively affecting job satisfaction (58). These factors collectively contribute to the decline in job satisfaction among experienced pharmacists. This also demonstrates that job satisfaction is not caused by a single factor, but is multidimensional and requires improvement from multiple aspects in order to ultimately enhance pharmacists’ job satisfaction.

### 4.7 Limitations and future research

Despite the strengths of our research, several limitations should be acknowledged, which also suggest potential directions for future research. Firstly, the use of convenience sampling limits the generalizability to the wider pharmacist population. The cross-sectional design also limits the ability to draw causal inferences. Additionally, the sample was limited to pharmacists working in private medical institutions in Guiyang city, meaning that the findings may not be applicable to other regions or settings. Data collected from different locations or types of institutions may yield different results. Secondly, the sample size was relatively small, which suggests that future research should involve larger and more diverse samples. Furthermore, reliance on self-reported questionnaires introduces the risk of social desirability and recall bias, which could affect the accuracy of the data. Moreover, our research focused solely on three dimensions—psychological contracts, job burnout, and job satisfaction—leaving out other potentially important factors, such as turnover intention or qualitative research methods (e.g., interviews), which could provide deeper insights. Lastly, the observed mediation effect (12.16%) was small, with limited practical relevance. Therefore, future research could build on these findings by exploring these additional factor in greater detail.

## 5 Conclusion

The research findings indicate that the psychological contract of pharmacists in private medical institutions in Guiyang is significantly weakly negatively correlated with job burnout and significantly strongly positively correlated with job satisfaction. There is a significant strongly negatively correlated between job burnout and job satisfaction. Mediation analysis indicates that job burnout serves as a weak mediator between the fulfillment of the psychological contract and job satisfaction. Thus, psychological contract fulfillment and job burnout are important factors affecting job satisfaction in private medical institutions. It is recommended that private medical institutions take proactive measures to ensure the stability of pharmacists’ psychological contracts. For example, during the intervention process, preventive guidance should be provided to senior pharmacists with longer tenures to avoid breaches of the psychological contract and the continued development of job burnout, thereby improving pharmacist satisfaction. For newly hired pharmacist, selective preventive interventions should be implemented, and management should be conducted according to the expected level of psychological contract fulfillment, achieving personalized psychological contract interventions. Furthermore, to avoid long-term fixed work patterns, implementing reasonable compensation and performance-based rewards can help reduce job burnout. Performance should be allocated based on the specific tasks undertaken by pharmacists, with dynamic adjustments to performance levels, rather than using fixed performance metrics to evaluate their work content and intensity. Lastly, this research helps to fill the research gap regarding pharmacists’ psychological contracts in private medical institutions.

## Data Availability

The original contributions presented in the study are included in the article/supplementary material, further inquiries can be directed to the corresponding author.
